# Dynamic and differential regulation in the microRNA expression in the developing and mature cataractous rat lens

**DOI:** 10.1111/jcmm.12094

**Published:** 2013-07-11

**Authors:** Eri Kubo, Nailia Hasanova, Hiroshi Sasaki, Dhirendra P Singh

**Affiliations:** a Department of Ophthalmology, Kanazawa Medical UniversityIshikawa, Japan; b Department of Ophthalmology and Visual Sciences, University of Nebraska Medical CenterOmaha, NE, USA

**Keywords:** microRNA, lens development, cataract, tropomyosin, ageing

## Abstract

Recent evidence supports a role for microRNAs (miRNAs) in regulating gene expression, and alterations in gene expression are known to affect cells involved in the development of ageing disorders. Using developing rat lens epithelial cells (LECs), we profiled the expression of miRNAs by a microarray-based approach. Few gene expression changes known to be involved in pathogenesis or cytoprotection were uniquely influenced by miRNA expression. Most miRNAs increased or decreased in abundance (let 7b, let 7c, miR29a, miR29c, miR126 and miR551b) in LECs/lenses during late embryonic and post-natal development and in cataract. Among them, miR29a, miR29c and miR126 were dramatically decreased in cataractous LECs from Shumiya Cataract Rats (SCRs). Specifically, the cytoskeleton remodelling genes tropomyosin (Tm) 1α and 2β, which have been implicated in the initiation of pathophysiology, were targets of miR29c and were over-stimulated as demonstrated by inhibitor experiments. In transfection experiments, increasing the level of miR29c caused a corresponding decrease in the expression of Tm1α and 2β, suggesting that miR29c may regulate the translation of Tm1α and 2β. 3′UTR luciferase activity of Tm1α, not 2β, was significantly decreased in miR29c-transfected mouse LECs. These findings demonstrate changes in miRNAs expression, and target molecules have potential as diagnostic indicators of ageing and as a foundation of miR-based therapeutics for age-related diseases.

## Introduction

Accumulating evidence indicates that both transcriptional and post-translational regulation mechanisms are pivotal in governing cellular fate and thereby determining developmental progression and physiological status in animals. Ageing, a highly complex phenomenon, is characterized by an increase in age-associated degenerative diseases such as type 2 diabetes, and neurological and cardiovascular diseases including blinding disorders. The age-associated blinding disease cataract has been suggested as a unique and elegant model for studying the development and progression of ageing [1–5]. Many processes, such as development, senescence, cellular signalling and genome maintenance are known to be regulated by miRNAs, which acts by controlling gene expression [6–9]. However, several adaptive mechanisms in cells alter gene function by regulating their expression in response to cellular requirements. These mechanisms include fundamental cellular processes such as cell cycle progression, gene transcription and translation, post-translational modifications and epigenetic remodelling. With the discovery of miRNAs and small interfering RNAs (siRNAs), the importance of post-translational gene regulation has become widely recognized. Regulation of gene expression by miRNAs is thought to be crucial for cellular integrity and organogenesis [9]. The miRNAs are a class of small, non-coding RNAs, ∼23 nucleotides that recognize target mRNAs by base pairing and thereby regulate their expression post-transcriptionally [10, 11]. To date, more than 3% of the genes in humans have been found to encode for miRNAs. Anywhere from 40% to 90% of the human protein-encoding genes are under miRNA-mediated gene regulation [12]. Although the precise mechanism of miRNA-mediated gene silencing remains uncertain, miRNAs can promote de-adenylation that likely destabilizes mRNAs, leads to their clearance and/or induces translational repression of target mRNAs [13–16]. Recent advances demonstrate that miRNAs play important roles in developmental timing, differentiation, cell proliferation and apoptosis [10, 11, 17–20]. Although some subsets of miRNAs are specifically expressed during early mammalian development [18], others are essential for morphogenesis of particular organs such as brain [20] and heart [21], or for hematopoietic differentiation [22]. Extensive studies conducted in *Caenorhabditis elegans* have shown that miRNAs play a role in determining lifespan [23], and a majority of the age-regulated miRNAs were found to be down-regulated in older animals. These data suggest that miRNA expression is reduced during ageing.

Recent investigations have shown that changes in miRNA levels also occur during cellular senescence *in vitro*, and the changes are associated with at least some aspects of ageing or age-associated degenerative disorders [24, 25]. Furthermore, single tissue-specific miRNAs have been identified in specific mammalian cell types or organs [26]. Although it has been reported that brain-specific miR-124, miR-125b and let-7 are expressed in mouse and rat eye lenses [27–30], no studies have involved the spatial and temporal expression profiles of miRNAs in lens development and cataractogenesis.

Moreover, a decline in expression and activity of naturally occurring antioxidants, leading to cellular malfunction and vulnerability in response to oxidative stress during ageing, has been shown experimentally to be a major cause in the initiation of pathophysiology of tissues and progression of several degenerative diseases including cataractogenesis [1, 5, 31–35]. Antioxidant reduction leads to accumulation of reactive oxygen species (ROS), a major cause of cell loss in the eye lens. ROS affect many cellular functions by damaging DNA, oxidizing proteins and causing lipidperoxidation [33, 36], which proceeds to derangement of structural cytoskeleton proteins such as actin, catherin and tropomyosins (Tm) that are essential to maintaining unique structures and configurations of cells [37].

The eye lens, an epithelial tissue derived from surface ectoderm, is composed of three principal cell types: central lens epithelial cells (LECs), germinative zone epithelial cells and fibre cells. Lens epithelial cells are located on the anterior surface of the lens as a monolayer, and lens fibre cells comprise the rest of the lens. Progenitors of lens fibre cells, LECs differentiate into lens fibre cells at the lens equator [38]. In the human lens, most central LECs survive for decades without dividing or differentiating, but ∼14% of these cells die over the course of a 75-year lifetime, leaving gaps or areas covered by aberrant processes of adjacent cells in the central epithelial monolayer [39]. Fibre cells have an extremely elongated shape and align along the anterior–posterior lens axis. Their ordered, anterior–posterior alignment is required for lens transparency. Any disruption of this organization not only impairs light transmission but also affects lens function, causing cataract [40, 41]. Age-related cataract is among the most common chronic disorders of ageing and is the world's leading blinding disorder. Thus, there is a pressing need to understand the basic mechanism underlying regulation of gene expression at physiological levels. We believe that clues to potential development of miRNA-based therapy for correcting or delaying lens abnormalities may be found by spotting out the genes that are regulated by a subset of miRNAs and that possibly play roles in lens development and differentiation, and investigating how their expression levels affect lens physiology.

In the study presented here, we have profiled miRNA expression during prenatal and post-natal development as well as in cataractous and non-cataractous LECs isolated from Shumiya Cataract Rats (SCRs), a model for hereditary cataract. We surveyed mRNA, miRNA and protein expression changes, determining spatial and temporal patterns of miRNA expression. This study should yield insight into the biological functions of miRNAs for lens development, differentiation and cataractogenesis. In addition, we investigated miR 29c regulation of the cytoskeletal structural proteins Tm1α and 2β, whose aberrant expression has been implicated in the initiation of pathophysiology. Alterations in the expression levels of miRNAs and their predicted targets that occur during embryonic development and cataract formation may serve as biomarkers for embryonic developmental stages as well as cataractogenesis, and may provide a basis for the development of agents that will postpone such disorders.

## Materials and methods

### Animals

All animal experiments were approved by the Committee of Animal Research at the Kanazawa Medical University, Japan (Permission number: 88), and were conducted in accordance with the National Institutes of Health's Guide for the Care and Use of Laboratory Animals and the Institutional Guidelines for Laboratory Animals. We used Sprague-Dawley albino rats with the following ages: embryonic day (ED) 16, post-natal 4-week-old (4W) and 14W. The rats were obtained from a local animal dealer (Clea, Osaka, Japan). Shumiya Cataract Rats at 12W were used as an animal model for cataract. Approximately, 70% of SCRs develop cataract from ∼11 to 13W onward [42]. The SCRs were divided into two groups, cataractous (SCR with cataract) and non-cataractous (SCR without cataract) using slit lamp microscope at 11 weeks of age.

### Cell culture

Primary cultured LECs were isolated from 6W BalbC mice (*n* = 8) and 8W SCRs with cataract and SCRs without cataract (*n* = 8). Lens epithelial cells were maintained in Dulbecco's Modified Eagle's Media (DMEM; Invitrogen, Carlsbad, CA, USA) with 10% foetal bovine serum (FBS; Sigma-Aldrich, St. Louis, MO, USA) as described previously.

### RNA extraction from lens

Six rats from each group were killed with CO_2_. Each rat lens was isolated and removed. Total RNA from each LEC with capsule was extracted with TRIzol method (Invitrogen), according to the manufacturer's protocol. Samples of RNA were set aside for real-time PCR to verify the results obtained from the microarray analysis.

### miRNA microarray

A new, sensitive, accurate and multiplexed miRNA profiling assay was performed. The assay is based on a highly efficient labelling method that uses Agilent miRNA labelling Reagent Kit (Agilent Technologies Inc., Santa Clara CA, USA) and a novel microarray probe design that uses Agilent Rat miRNA microarray containing unique gene probes for 349 miRNAs. Using a simple, single-vial experimental protocol, 100 ng of total RNA was directly labelled using Cy3, without fractionation or amplification. The labelled miRNAs were combined with 4.5 mg of random DNA 25-mers (Operon Biotechnologies, Tokyo, Japan). Each sample was hybridized onto a microarray at 55°C for 20–48 hrs. Slides were scanned on an Agilent DNA microarray scanner. Agilent Feature Extraction software version 8.1 was used for image analysis.

### Statistical and bioinformatics analysis of microarray data

Microarray data were analysed using Agilent Gene Spring GX software. Per chip normalization was done by dividing each gene's measurement by the specific control measurements or by the average intensity in the single array. Normalized data were exported for subsequent analysis. Genes with normalized ratio more than 2.0-fold or less than 0.5-fold were selected as significant genes among three samples.

### Real-time PCR

Quantitative RT-PCR validation was performed with TaqMan® MicroRNA RT kit and TaqMan® MicroRNA assays (Applied Biosystems, Foster City, CA, USA) following the manufacturer's protocol. Reactions were performed on an ABI PRISM® 7300 thermocycler (Applied Biosystems), and cycle threshold values were determined by the manufacturer's software. Comparative CT methods were used for relative quantification of miRNA expression. We performed three independent experiments for each assay. Values are expressed as means ± SD.

### Transfections of miRNA inhibitors and precursors

For inhibition of miRNAs, miRIDIAN Hairpin inhibitor (Thermo scientific, Lafayette, CO, USA), Rat rno-miR29a and 29c were used and transfected into SCRs with cataract and SCRs without cataract LECs. For overexpression of miRNAs, miR-mR29a and 29c miRNA precursors (Applied Biosystems) were used and transfected into LECs of SCRs with cataract. Both transfections were performed with the electroporation method using the Neon™ Transfection system and following the manufacturer's protocol (Invitrogen). We performed three independent experiments for each assay. Values are expressed as means ± SD.

### Protein expression analysis

Protein lysates of transfected LECs were prepared in ice-cold radioimmune precipitation buffer, and protein blot analysis was performed as described previously [33, 35, 43]. The membranes were probed with anti-Tm monoclonal antibody (TM311; Acris Antibodies, Herford, Germany) (dilution 1:500), which recognizes Tm1α and 2β. For visualization, these membranes were incubated with horseradish peroxidase-conjugated secondary antibodies. Specific protein bands were visualized by incubating the membrane with luminal reagent (Roche, Tokyo, Japan) and the images were recorded with ImageQuant LAS-4000 luminescent image analyser (GE Healthcare Japan Corporation, Tokyo, Japan). To ascertain comparative expression and equal loading of the protein samples, the membrane stained earlier was stripped and re-probed with β-actin antibody (Sigma-Aldrich). We performed three independent experiments for each assay. Values are expressed as means ± SD.

### 3′-UTR luciferase assay

A 779-bp fragment of rat TM1α3′-UTR and a 291-bp fragment of rat TM2β 3′-UTR inserted into a pEZX-MT01 vector with fLuc reporter gene and miRNA Target clone control vector for pEZX-MT01 were designed and purchased from GeneCopoeia™ (GeneCopoeia Inc., Rockville, MD, USA). Primary cultured mouse LECs were plated in 96-well plates. Rat TM1α3′-UTR, rat TM2β 3′-UTR was cotransfected with miExpress™ Precursor miRNA Expression Clone for rno-miR29a mimic or rno-miR29c mimics in pEZX-MR04 vector with eGFP reporter gene, Precursor miRNA scrambled control for pEZX-MR04 or empty pEZX-MR04 vector purchased from GeneCopoeia Inc. Luciferase assays were performed three times 48 hrs after transfection using Luc-Pair™ miR Luciferase Assay (GeneCopoeia Inc.). Firefly luciferase activity was normalized to Renilla luciferase activity and expressed as% maximum stimulation relative to control vector–transfected cells. Results shown are averages of six separate experiments performed in triplicate. We performed three independent experiments for each assay. Values are expressed as means ± SD.

### Statistical methods

The correlation between gene expression levels was analysed using one-factor anova, with data expressed as mean ± SD. Differences were considered statistically significant at *P* < 0.05. All analyses were performed with StatView ver. 5.0 (SAS Institute Inc., Cary, NC, USA).

## Results

### Changes in miRNA expression profile in progression of eye lens development

**Fig. 1:**
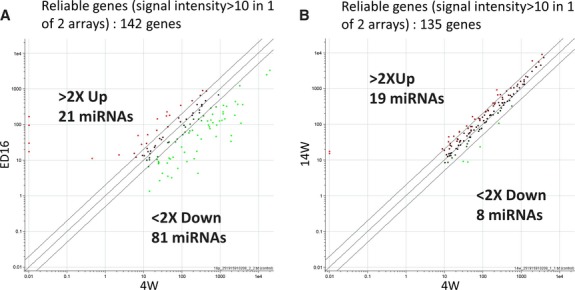
Scatter plot analysis. (**A**) Scatter plot matrix of each miRNA array for the ED16 (*y*-axis) and 4W (*x*-axis) rat lenses showing mean intensities in the reference channel. (**B**) Scatter plot of miRNA expression in 14W (*y*-axis) and 4W (*x*-axis) lenses. The scatter plots show representative data, using mRNA from rat lens epithelial cells. Each dot represents one gene. Reliable genes (signal intensity >10 in array); 142 genes (**A**) and 135 genes (**B**). A twofold change was considered to provide reliability of gene expression.

In analysing the expression levels of miRNAs at different stages of lens development (prenatal ED16, and post-natal 4W and 14W), we detected expression signals from a total of 100 differentially regulated miRNAs (Tables 1 and 2) at a cut-off >10.0 (*P* ≤ 0.05, Bonferroni's correction for multiple testing). Of these genes, 56 miRNAs were up-regulated in 4W and 14W lenses in comparison with prenatal ED16 lenses (Table 1), in which 44 miRNAs were found to be down-regulated (Table 2). Collectively, data suggested involvement of these genes in the progression of development. Figure 1, scattergrams of mRNA expression, illustrates the differences between the control group (4W) and groups ED16 and 14W. However, altered expression of the majority of genes was within the twofold range (Fig. 1). Notably, in group ED16, expression of 21 miRNAs was significantly increased, and expression of 81 miRNAs was significantly decreased in comparison with the 4W group of lenses. In group 14W, the expression of 19 miRNAs was significantly up-regulated and expression of 8 miRNAs was down-regulated significantly compared with group 4W. The data should be helpful in predicting changes in miRNA expression during early ageing of lens. Any significant perturbations to normal profiles of miRNA expression during early development can be early indicators of future phenotypic changes, such as cataract or at least aberrant lens development.

**Table 1:** Expression levels of 56 miRNAs were up-regulated (twofold) in 14W *versus* ED16 and in 4W *versus* ED16 lenses

Group	ED16	4W	14W

miRNA	Normalized ratio	Normalized ratio	Normalized ratio
rno-miR-374	1	11.913	15.304
rno-miR-148b-3p	1	4.860	5.722
rno-miR-27a	1	6.103	7.497
rno-miR-210	1	8.017	10.150
rno-miR-31	1	57.292	90.646
rno-let-7i	1	8.418	9.932
rno-miR-181a	1	2.527	2.7451
rno-miR-30e	1	3.2337	3.599
rno-let-7e	1	3.537	3.801
rno-miR-128	1	4.560	5.939
rno-miR-652	1	2.602	3.077
Tio-niiR-181c	1	2.397	2.788
rno-let-7f	1	13.347	22.336
mo-miR-872	1	9.729	12.997
rno-miR-30a	1	6.033	7.850
rno-let-7d	1	16.821	25.418
rno-miR-125a-5p	1	2.106	2.339
rno-miR-29b	1	16.649	51.422
rno-miR-101b	1	4.976	9.279
rno-miR-29c	1	13.614	37.460
rno-let-7b	1	39.675	121.754
rno-miR-125b-5p	1	7.7633	14.711
rno-miR-184	1	6.488	11.696
rno-miR-204	1	5.999	10.187
rno-miR-130a	1	2.660	3.657
rno-miR-23b	1	6.940	13.240
rno-miR-140*	1	4.151	6.664
rno-miR-101a	1	7.002	13.307
rno-let-7c	1	31.570	82.739
rno-miR-24	1	5.7109	9.288
rno-miR-27b	1	5.107	8.084
rno-miR-384-5p	1	2.008	2.433
rno-miR-186	1	2.114	2.571
rno-miR-425	1	10.287	18.866
rno-let-7a	1	10.6657	19.893
rno-miR-26a	1	5.751	9.208
rno-miR-26b	1	5.258	7.720
rno-miR-99a	1	8.3438	13.599
rno-miR-338	1	14.7728	27.0430
rno-miR-22	1	9.178	15.166
rno-miR-100	1	18.881	35.391
rno-miR-361	1	12.971	24.674
rno-miR-30b-5p	1	3.723	5.17405
rno-miR-23a	1	7.652	12.554
rno-miR-221	1	2.943	3.799
rno-miR-30c	1	4.567	6.570
rno-miR-125a-3p	1	3.077	7.574
rno-miR-29C*	1	2.1587	3.648
rno-miR-151	1	2.761	4.711
rno-miR-211	1	2.5078	4.324
rno-miR-487b	1	2.651	6.823
rno-miR-218	1	5.054	4.788
rno-miR-333	1	4.005	3.780
rno-miR-96	1	60.873	24.561
rno-miR-182	1	7.133	2.129
rno-miR-124	1	4.281	2.424

Up-regulation of a set of 56 miRNAs was observed in 4W and 14W lenses relative to ED16. These were derived following statistical analysis as stated in Materials and methods. The table was sorted by amount of change in 4W and 14W if ED16 normalized value is 1. miRNAs showing more than twofold relative expression were incorporated in the table.

**Table 2:** Forty-four miRNAs were down-regulated in 4W and 14W lenses relative to ED16 prenatal rat lenses

Group	ED16	4W	14W

miRNA	Normalized ratio	Normalized ratio	Normalized ratio
rno-miR-320	1	0.01	0.01
rno-miR-378	1	0.01	0.01
rno-miR-183	1	0.01	0.01
rno-miR-130b	1	0.01	0.01
rno-miR-365	1	0.01	0.01
rno-miR-140	1	0.01	0.01
rno-miR-192	1	0.01	0.01
rno-miR-664	1	0.01	0.01
rno-miR-98	1	0.01	0.01
rno-miR-190	1	0.01	0.01
rno-miR-195	1	0.01	0.01
rno-miR-127	1	0.01	0.01
rno-miR-194	1	0.01	0.01
rno-miR-216a	1	0.01	0.01
rno-miR-18a	1	0.01	0.01
rno-miR-30e*	1	0.01	0.01
rno-miR-340-5p	1	0.01	0.01
rno-miR-30c-2*	1	0.01	0.01
rno-miR-10a-5p	1	0.01	0.01
rno-miR-499	1	0.01	0.01
rno-miR-352	1	0.01	0.01
rno-miR-497	1	0.01	0.01
rno-miR-223	1	0.01	0.01
rno-miR-152	1	0.01	0.01
rno-miR-339-3p	1	0.01	0.01
rno-miR-185	1	0.01	0.01
rno-miR-30a*	1	0.01	0.01
rno-miR-500	1	0.01	0.01
rno-miR-29a	1	0.01	0.01
rno-miR-204*	1	0.01	0.01
rno-miR-20a	1	0.385	0.379
rno-miR-466b	1	0.314	0.291
rno-miR-19a	1	0.264	0.243
rno-miR-92a	1	0.461	0.434
rno-miR-672	1	0.145	0.177
rno-miR-17-5p	1	0.269	0.225
rno-miR-22*	1	0.021	0.01
rno-miR-301a	1	0.224	0.163
rno-miR-206	1	0.040	0.01
rno-miR-451	1	0.180	0.039
rno-miR-126	1	0.157	0.01
rno-miR-136	1	0.159	0.01
rno-miR-329	1	0.364	0.047
rno-miR-551b	1	0.397	0.01

Statistical analysis of microarray data revealed that a set of miRNAs was down-regulated significantly in post-natal lenses (4W and 16W) relative to ED16. The number 44 down-regulated miRNAs was determined on the basis of twofold or greater changes. Extreme left vertical column, group of miRNAs is followed by ED16 as normalization control, 4W and 16 W, showing down-regulated ratio.

To address whether the changes in miRNA expression levels may have been associated with developmental stages, we analysed levels of miRNAs statistically and listed those that were up-regulated twofold (ED16<4W<14W) or down-regulated (14W<4W<ED16) progressively during developmental and post-natal stages (Table 3). Data revealed consistent altered expression in miRNA levels; in this context, we propose that expression profile in miRNAs may serve as a marker of eye lens development and may be useful in predicting the onset of adverse changes. The predicted targets for each miRNA by Sanger miRBase Software may potentially reveal diseases, molecular functions, pathophysiological and developmental disorders, and canonical pathways associated with each miRNA (Table 4).

**Table 3:** Statistical analysis of microarray data showing post-natal twofold down- or up-regulation of miRNAs

Gene name	ED16	4W	14W
2XDown			
rno-miR-126	1	0.157	0.01
rno-miR-136	1	0.159	0.01
rno-miR-206	1	0.040	0.01
rno-miR-329	1	0.364	0.047
rno-miR-451	1	0.180	0.039
rno-miR-551b	1	0.397	0.01
2XUp			
rno-let-7b	1	39.675	121.754
rno-let-7c	1	31.570	82.739
rno-miR-29b	1	16.649	51.422
rno-miR-29c	1	13.614	37.460
rno-miR-204*	1	5.999	10.186

Table summarizes the relative changes in expression levels of miRNAs in lenses of rats after birth. Statistical analysis disclosed that 6 miRNAs were down-regulated (upper panel) and 5 miRNAs were up-regulated (lower panel) significantly.

**Table 4:** Description of putative target genes (from rat genome) associated with lens development and cataractogenesis

	ED16>4W>14W
2XDown	
miR-126	NE-kappa-B inhibitor alpha (l-kappa-B-alpha), Insulin-like growth factor–binding protein 7 (IGFBP-7), Bcl2 antagonist of cell death (BAD), Bcl-2-like protein 11, Tumor protein p53-inducible nuclear protein 1, Mitogen-activated protein kinase 9 (Stress-activated protein kinase JNK2), Thioredoxin reductase 1, Novel EGF-like domain-containing protein, Collagen alpha-1(XIV) chain precursor (Undulin), SPARC-related modular calcium binding 2
miR-136	IGFBP-2, Multidrug resistance protein 2, 78 kD glucose-regulated protein precursor (GRP 78) (Heat shock 70 kD protein 5), Tropomodulin-4, EGF-like domain-containing protein 4
miR-329	Oxidative stress-induced growth inhibitor 1, Tumor protein p53-induclble nuclear protein 1, FGF receptor substrate 2, IGFBP-2, GRP-78, Mothers against decapentaplegic homolog 6(SMAD 6), EGF-like, fibronectin type III and laminin G domains isoform 3, wild-type p53-induced gene 1, FGF-10, Translation initiation factor elF-2B subunit beta (elF-2B), SMAD 2, Bcl-2-like protein 11, Tropomyosin alpha-3 chain (Tropomyosin-3), Heat shock 70 kD protein 12B, thioredoxin-related transmembrane protein 2, FGF-5
miR-451	Tropomodulin 3, EGF-llke-domain multiple 6 Gene, FGF22, tumour necrosis factor receptor superfamily member 1A precursor (pSO), Aquaporin-11 (AQP-11), SPARC-related modular calcium binding 2, Axin-1 up-regulated gene 1 protein (TGF-beta-induced apoptosis protein 3) (TAIP-3), GRP78, BCL2-like 12, EGF-like domain-containing protein 4, Multiple EGF-like domains 6 precursor, thioredoxin-related transmembrane protein 2
miR-551b	IGF2 mRNA-binding protein 1, elF-2B, FGF 8, Peroxiredoxin-6 (Antioxidant protein 2) (1-Cys peroxiredoxin), thioredoxin-related transmembrane protein 2, glutathione S-transferase M4, Platelet-derived growth factor (PDGF) receptor-like protein precursor, GRP 78, Proto-oncogene protein c-fos (Cellular oncogene fos), EGF-like protein 6 precursor, TGF beta-3 precursor (TGF beta-3) TGF-beta-induced protein ig-h3 precursor (Beta ig-h3), Cellular tumour antigen p53 (Tumor suppressor p53), procollagen, type IV, alpha 4, Glucocorticoid receptor (GR).
2XUp	
let-7b	poly (ADP-ribose) polymerase family, member 3, Glutaredoxin-2, Fibroblast growth factor 20 (FGF-20), FGF-binding protein 1, Multiple EGF-like domains 6 precursor, Hsp70-binding protein 1, IGF2 mRNA-binding protein 3, Myc proto-oncogene protein (c-Myc), Hsp70-binding protein 1 (HspBP1), FGF receptor activating protein 1, Tumor protein p53-inducible nuclear protein 1, Vimentin, Thioredoxin mitochondrial precursor (Mt-Trx)
let-7c	poly (ADP-ribose) polymerase family, member 3, Vascular endothelial growth factor C precursor (VEGF-C), FGF-20, IGF2 mRNA-binding protein 3, Glutaredoxin-2, NF-kappa-B essential modulator (NEMO) (NF-kappa-B essential modifier), Hsp70-binding protein 1, c-Myc, Heat shock factor 2-bindlng protein, AQP-2, Tumor protein p53-inducible nuclear protein 1, Vimentin, Mt-Trx
miR-29a	Tropomyosin alpha-1 chain (Tropomyosin-1) (Alpha-tropomyosin), glutathione peroxidase 7, PDGF B-chain, Dicer1
miR-29c	Tropomyosin-1, Dicer1, TGF-beta-2, glutathione peroxidase 7, PDGF A-chain, Multiple EGF-like domains 6 precursor, FGF receptor–activating protein 1, SMAD 6, AQP-11
miR-204*	Tropomyosin-1, Hsp70-binding protein 1, Mitogen-activated protein kinase 4, Gamma crystallin D, IGFBP-1, Glutathione S-transferase P (GST class-pi), Sulfiredoxin 1

To predict genes targeted by miRNAs, Sanger miBase Software was utilized. Originally hundreds of transcripts containing multiple predicted miRNA target sites were detected. We enhanced the stringency for prediction of sites by applying highest context score filter. This reduced the number of target genes significantly, and a short list of the most potentially applicable putative genes appears in the table.

### Confirmatory assays with differentially expressed miRNAs by RT-PCR

**Fig. 2:**
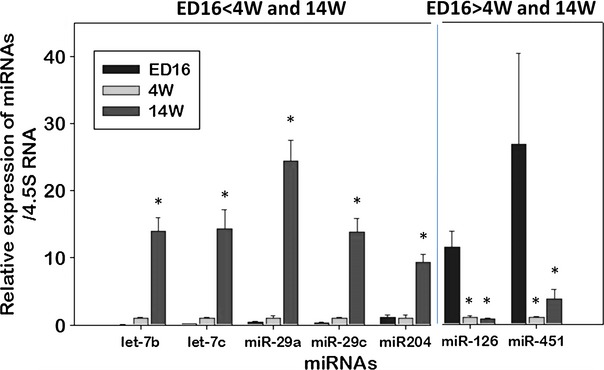
RT-PCR validation of relative changes in miRNA expression in lenses from ED16, 4W and 14W rats. Expression of selected and functionally significant miRNAs was further validated using RT-PCR as described in Materials and methods. Relative expression levels of miRNAs, let-7b, let-7c, miR-29a, miR-29c, miR-204, miR-126, miR-451 in ED16, 4W and 14W lenses are represented as histograms with normalized averages ± SD. *P < 0.01 or 0.0005 by Student's *t*-test.

The results of microarray presented above showed the modulation in expression levels of several miRNAs during progression of eye lens development, indicating the importance of their regulatory roles in gene expression during development. However, we observed that miRNAs belonging to the same class/family have similar expression profiles, suggesting that they may not be differentiated in microarray analysis because of the possibility of cross-hybridization. To avoid this, we selected functionally known and well-defined miRNAs whose expression was modulated in our microarray data, and validated their expression by RT-PCR (Fig. 2). Figure 2 shows significant modulation in these miRNAs during the course of development. The seven miRNAs (let7b, 7c, miR29a, miR-29c, miR204, miR126, miR51) listed in Table 3 were used for validation experiments.

Next, we analysed data to precisely identify any alteration in temporal or spatial expression of these miRNAs through progression of development. Levels of let-7b, let-7c, miR-29a, miR-29c and miR-204 were dramatically altered during the progression of development. In contrast, miR126 and miR-451 were significantly down-regulated (Fig. 2). The data emphasized the variability in miRNA expression over time, which may be part of an important biological process involving the time-dependent expression of genes within the cellular microenvironment. These miRNAs may be involved in tight regulation of lens development by controlling the gene expression. However, after analysis and evaluation with the miRNA target gene evaluation programme [44], coupled with the genes’ known functions, we surmised that these miRNAs were associated with various target genes (Table 4), including cytoskeleton proteins Tm1α and 2β, in which aberrant expression has been shown to damage cellular integrity and to be involved in both cancer and ageing [45–47].

### Cataractous and non-cataractous lenses or LECs from SCRs revealed significantly altered expression of miRNAs

**Fig. 3:**
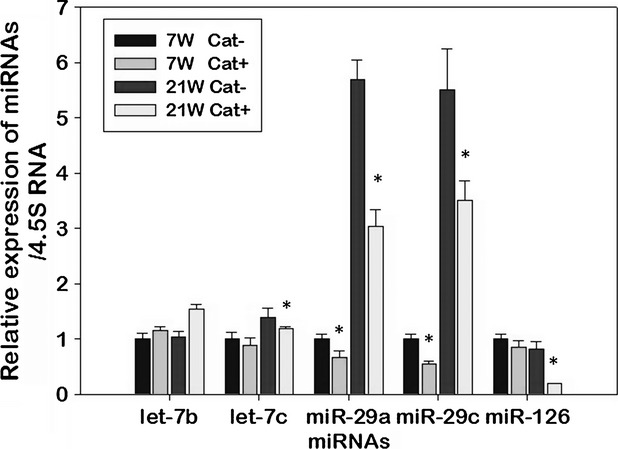
Analysis of changes in miRNA expression in lenses from Shumiya Cataract Rats (SCR) with cataract or without cataract. Relative expression of miRNAs let7b, let7c, miR-29a, miR29c and miR126, which are significantly up- or down-regulated during the progression of lens development, were measured following RNA extraction using RT-PCR. Histograms represent normalized mean results ± SD. *P < 0.05 or *P* < 0.001 values are considered statistically significant compared with control. Internal control was taken to evaluate integrity of RNA. Data revealed that let7c, miR29a and miR29c were significantly up-regulated in 21W lenses compared with 7W SCR without cataract (Cat−) or SCR with cataract (Cat+). However, let7c, miR-29a, miR29c and miR126 were significantly down-regulated in SCR with cataract lenses compared with SCR without cataract lenses at both ages 7 and 21 weeks.

Recent studies have suggested that changes in miRNA expression levels occur during the initiation or progression of several diseases. Research using an *in vitro* model system has shown that the onset of cellular abnormalities involves an irreversible decline in cell proliferation, and such cells display significant modulation of miRNAs [48]. In addition, recently, a generally altered expression in miRNA levels, with some exceptions, has been observed [7, 8]. To examine whether miRNA expression differs in cataractous and non-cataractous LECs, we conducted RT-PCR with RNA isolated from cells or lenses from non-cataractous and cataractous rats using specific probes for selected miRNAs. As shown in Figure 3, Let 7c, miR-29a and miR29c were up-regulated in non-cataractous LECs/lenses compared with cataractous. However, expression of these miRNAs and miR126 was decreased in SCRs with cataract. Importantly, when we analysed expression changes separately in development and in SCRs with and without cataract, we found putative similar regulatory effects of some miRNAs, but modulation of expression was more significant in the disease state. These data suggest that down-regulation of let7c, miR29a and miR29c, and miR126 may be a major event during cataract formation in the SCR, as changes in miRNA expression levels were increased in cataractous lenses. We propose that expression of these miRNAs and their putative target genes is an indicator of pathogenesis, as well as a potential target for the development of therapeutic molecules.

### Prediction of target genes of miRNAs and pathways related to lens development and cataractogenesis

The functional characterization of miRNAs relies largely on identification of their target mRNAs. Different miRNAs can regulate the same target genes, or each individual miRNA can regulate many target genes, providing great potential for multiple or overlapping signalling pathways. However, the major problem for current miRNA research is defining the biologically relevant and functional targets that they regulate. Several bioinformatics tools and experimental approaches have been used for spotting out putative target miRNAs or their target mRNAs [14, 16]. To explore what targets and pathways may be regulated by the miRNAs identified in this study as developmentally expressed or expressed during cataractogenesis, we utilized Sanger miBase Software. Table 4 shows the target genes (from rat genome) for the cloned miRNAs that are possibly related to genes for lens development and cataractogenesis. We realize, however, that these detected miRNAs may control many genes. We found that most of the predicted target genes that may be regulated by the miRNAs belonged to oxidative stress- and antioxidant-associated signalling, transforming growth factor (TGF) βs or fibroblast growth factors (FGFs) and platelet-derived growth factors (PDGFs; Table 4). These factors have been shown to play a pivotal role in lens development [4, 49, 50]. Importantly, modulated expression or activation of the growth factors by oxidative stress leads to cellular abnormalities and cataractogenesis [49, 50]. We also found that a target of miR 551b was peroxiredoxin (Prdx)6, a multifunctional protein essential for cell proliferation, differentiation and protection, whose deficiency causes cell death and cataractogenesis and whose aberrant expression results in tumorigenesis [1, 32, 34, 43, 51, 52]. Decreased levels of Prdx6 have been shown to cause activation of expression of TGFβs [1] that leads to extracellular matrix protein up-regulation [1, 32, 34, 43]. On the basis of the above results, we believe that developing agonists or antagonists that can govern miRNA levels should lead to an effective means of controlling disease evoked by overstimulation of miRNA-regulated genes.

Most importantly, the predicted target genes for miRNAs also included the cytoskeleton remodelling protein tropomodulin (target for miR451) and Tm 1α and 2β, the latter of which is a molecule essential for maintaining structural biology of several organs including eye lens [53]. Aberrant expression or ablation of this molecule has been shown to result in pathophysiology of tissues/organs [37, 53]. Our study revealed that Tm 1α and 2β are putative target genes for miR29a or 29c. However, it was intriguing to find that a majority of the predicted target genes for miRNAs identified in this study were related to extracellular matrix protein genes, and the pathways involved in this process included signalling mediated by TGF βs, FGFs and PDGFs [34, 50, 54, 55]. These factors have been found to mediate lens organogenesis, cell proliferation, apoptosis and cataractogenesis.

### Effect of miR29a and 29c inhibitors on the expression of Tm1α/2β levels

**Fig. 4:**
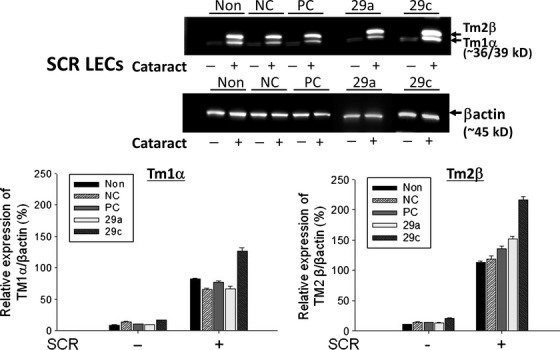
Elevated expression of Tm1α/2β proteins in lens epithelial cells (LECs) transfected with miR29a and 29c inhibitors. Lens epithelial cells isolated from Shumiya Cataract Rats (SCR) with cataract (+) or SCR without cataract (−) were cultured and processed for transfection with miRNA inhibitors as described in Materials and methods. At a pre-defined time, cellular extracts were prepared and subjected to protein expression analysis using antibody specific to Tm1α/2β. Immunoblotting experiments revealed greater abundance of Tm1α/2β protein in LECs transfected with miR29a and 29c inhibitor. Representative immunoblots show modulated expression of specific miRNA targeted gene Tm1α/2β. Blotted membranes were probed with antibody specific to Tm1α/2β and reprobed with β-actin antibody as internal and protein loading control. Histograms represent normalized mean results ± SD. *P < 0.05 is considered to be statistically significant. Non: No transfection; NC: Negative control; PC: Positive control.

From the predicted data (Table 4) showing the prevalence of oxidative stress and growth factors pathways that directly or indirectly affect cytoskeleton-associated protein expression, we envisage that optimum regulation of extracellular matrix or cytoskeleton protein should be a pivotal factor during normal progression of development. Aberrant expression and therefore altered activities may initiate abnormal signalling that may in turn proceed to a disease state. As extracellular or cytoskeletal molecules are thought to be important in maintaining normal development, and their abnormal expression may cause pathogenesis, we chose Tm1α and 2β molecules, predicted targets of the miR29 family and used Western blot analysis to examine their protein expression in cell extracts isolated from LECs transfected with miR29a or 29c inhibitors (Fig. 4). In most cases, miRNAs down-regulate the expression of their target genes/protein by inhibiting protein translation [56]. In protein blot, delivery of miR29c inhibitor to LECs significantly enhanced the abundance of Tm2β protein (Fig. 4). However, levels of Tm2β protein were originally up-regulated in cataractous LECs compared with control (non-cataractous LECs), suggesting that Tm2β was a target. miR29c affects Tm2β by modulating the Tm2β translation (Fig. 4).

In parallel experiments, we used miR29a inhibitor, and found it ineffective compared with miR29c. Furthermore, protein blot assessment of modulation of Tm1α expression revealed that the translational level of Tm1α did not alter significantly in LECs treated with miR 29a inhibitor. Protein level further increased in cells following transfection with inhibitor of Tm1α, suggesting involvement of some alternative pathway(s) at translation levels between Tm1α and 2β (Fig. 4).

### Effect of miR29a, 29c and let7b overexpression on the expression of Tm1α/2β proteins

**Fig. 5:**
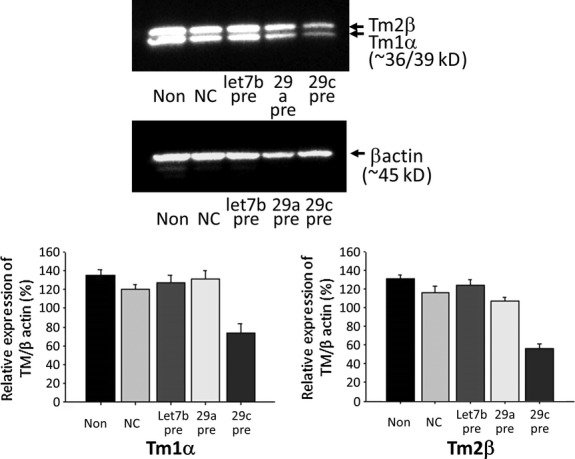
Lens epithelial cells (LECs) from Shumiya Cataract Rats (SCR) with cataract transfected with let7b, miR29a and 29c precursors display reduced expression of Tm1α/2β proteins. We used SCR with cataract LECs because their Tm1α/2β expressions were higher than those of SCR without cataract LECs. Cells were overexpressed with let7b, miR29a and miR29c by transfecting them with miRNA precursors. Immunoblotting experiments were conducted after processing of cellular extracts isolated from transfected cells. Blotted membranes were probed with antibody specific to Tm1α/2β and reprobed with β-actin antibody as internal and protein loading control. Immunoblots are representative of experiments and show reduced expression of Tm1α/2β protein in SCR with cataract LECs overexpressing miRNA 29c. Non: No transfection; NC: Negative control. Data are means ± SD from three independent experiments.

To further determine whether miR29a or miR29c regulates Tm1α/2β expression, we transfected LECs isolated from cataractous SCRs with precursors (pre) of these miRNAs. Figure 5 shows the expression levels of Tm1α/2β proteins in LECs transfected with miR29a and miR29c precursors isolated from SCR with cataract. Expression of Tm1α/2β protein was down-regulated in LECs following miR29c transfection, further demonstrating that miR29c may regulate and control Tm1α/2β gene expression. These findings suggest that aberrant expression of miR29c during progression of lens development or cataract formation may be one cause of cellular abnormalities that in turn result in abnormal cellular organization or defective organogenesis, leading to age-associated degenerative disorders including cataractogenesis.

### Direct regulation of miR29c to TM1α's transcripts

**Fig. 6:**
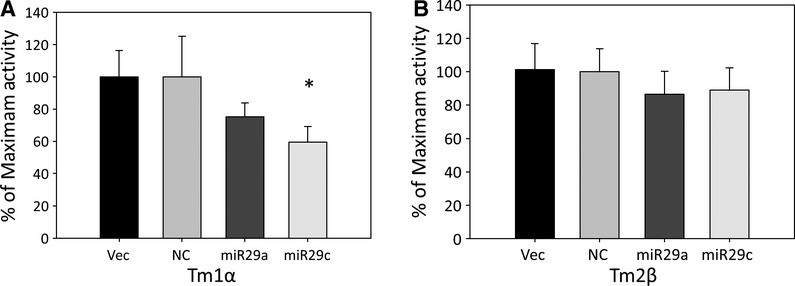
Luciferase reporter assay. Mouse lens epithelial cells were cotransfected with rat TM1α (**A**) or rat TM2β (**B**) with rno-miR29a mimic, rno-miR29c mimics in pEZX-MR04 vector with eGFP reporter gene, precursor miRNA scrambled control for pEZX-MR04 or empty pEZX-MR04 vector. The luciferase activity of firefly-luc and renilla-luc were measured at 48 hrs after transfection. The firefly-luc activity was normalized to Renilla-luc expression. Data are means ± SD from three independent experiments, each containing four replicates. *P < 0.02 values are considered statistically significant compared with vector control.

As shown in Figure 6A and B, when the luciferase gene carried 3′ UTR region of TM1α's transcript, the luciferase activity was inhibited by overexpressing miR29c compared with the negative control, but was not significantly inhibited by overexpressing miR29a (Fig. 6A). On the other hand, when the luciferase gene carried 3′ UTR region of TM2β's transcript, the luciferase activity was not significantly inhibited by overexpressing miR29a and miR29c compared with the negative control (Fig. 6B). These results suggest that Tm1α expression may be directly regulated by miR29c, not miR29a. Tm2β expression may be indirectly regulated by miR29c.

## Discussion

Gene expression is a fundamental process essential for maintenance of cellular phenotypes and cellular homeostasis.

Various cell-signalling effectors, such as transcription factors, cofactors and molecules related to pathways, are involved in controlling the expression levels of genes and gene products. It is now generally accepted that miRNAs regulate a variety of cellular pathways by regulating expression of multiple target genes [11].

miRNAs have roles in conditions associated with pathophysiology as well as stressor-induced cellular insults, including blinding diseases [7, 8, 57–59].

In this study, we performed microarray experiments to profile miRNA expression levels in the development of eye lens, and in LECs isolated from cataractous and non-cataractous SCRs. We showed, for the first time, that the expression of 100 miRNAs was significantly altered. An analysis of microarray data revealed that 44 miRNAs were up-regulated and 56 were down-regulated after birth in rat lens. Of the latter, miR126, miR136, miR206, miR451 and miR551b showed significant, gradual down-regulation (>2 times) after birth or during development (ED16>4W>4W). let7b, let7c, miR29b, miR29c and miR204 showed significant, gradual up-regulation (>2 times) after birth and during progression of lens development (ED16<4W<14W). These changes in miRNA expression levels were submitted to RT-PCR, which confirmed patterns of miRNA expression similar to those observed in microarray data. These data suggest a role for these miRNAs in the specification and/or progression of lens development in rats.

In previous research [27, 29, 30], several miRNAs such as miR124, miR7, miR125b and let7b have been detected in rat lens and in regeneration of new lens by transdifferentiation of pigment epithelial cells of the dorsal iris. Based on our results, we presume that developmental expression profiles are largely conserved (Table 1). In fact, similar expression changes during development are likely to be a progressive phenomenon that ends in a timely manner in favour of maintaining cellular integrity.

In other model systems, an antagonistic regulatory relationship between changes related to development and changes related to advancing of development has been proposed [60, 61]. Our data revealed that expression levels of miR7 and miR125b were unchanged in ED16, 4W and 14W lenses. These results argue against any significant role for these two miRNAs in progression of lens development. As a whole, our results reveal that expression dynamics and regulator interaction among the miRNAs and their targeted genes (Table 4) during early and late developmental stages are not independent. This finding is consistent with previous studies [8]. In addition, recent literature contains clear documentation showing that miRNAs play a role in every stage of progression of pathogenesis [6, 62–64]. Our study of the expression levels of miRNAs from cataractous and non-cataractous lenses isolated from SCR revealed changes in expression of specific miRNAs and their putative target gene(s) that may contribute to cataractogenesis (Fig. 3). Interestingly, RT-PCR assessment of level of miRNAs in cataractous lenses showed that four miRNAs–let7c, miR29a and 29c, and miR-126–were significantly down-regulated, suggesting a plausible contribution by these miRNAs to cataractogenesis (Fig. 3). These results are comparable to those of prior microarray studies in which a majority of miRNAs have been shown to be decreased [7, 48, 65]. Furthermore, our microarray data also showed up-regulated expression of miRNAs during the progression of lens development as well as in cataractous and non-cataractous ageing lenses at a certain time-point (Tables 1 and 3, Figs 1 and 2). This finding agrees with previously published studies in the mouse [66]. Li and associates showed specifically that 31 miRNAs were significantly up-regulated and 17 were down-regulated with advancing of brain development [66]. Taken together, these findings argue that up-regulation of specific miRNAs during development or ageing may target genes that are specific to specific cell/tissue types. The modulated expression of these miRNAs has been suggested as contributing in many cellular processes, including cellular abnormalities [8, 57, 67]. We hold the view that cataractogenesis may be the result of changes in the translational machinery because of changed expression of miRNA during the progression of cataract formation. Alternatively, this process may be essential for slowing the progression of development at some time-point, to maintain developmental homeostasis throughout life.

Moreover, miRNAs are known to exert their function primarily by influencing gene expression. miRNAs do so by base pairing with target mRNAs [16, 68]. miRNAs either inhibit translational initiation [16] or direct the target mRNA degradation [69]. It is not surprising that we did not achieve a strict inverse association between developmentally regulated miRNAs and their putative regulatory or regulated genes in our miRNA profiling. Table 4 shows that many genes potentially implicated in progression of development and cataract formation were found to be targets of miRNAs detected on microarray screen plot (Fig. 1, Table 4). To understand the significance and potential impacts of a change in the level of a certain miRNA in development or cataract formation, it is essential to identify the miRNA's target mRNA. However, computational predictions of miRNA target genes revealed the potential scope of gene regulation by miRNAs. We believe that the regulation of genes by significantly modulated miRNAs may involve the modulation of multiple genes/mRNAs. To identify possible roles of the miRNAs related to lens development and cataract, we examined the miRNA-regulated predicted target genes that were associated with eye lens, surveyed the literature [1, 33–35, 53, 70–94] and employed Sanger miBase software. As shown in Table 4, we found many target genes associated with those processes in the lens, such as TGF-βs and TGF-β-inducible genes related to extracellular matrix or cytoskeleton proteins such as βig-h3 precursor, vimentin, Prdx6, FGF and so on. Target gene prediction further showed the growth factor(s)-mediated cellular pathways involved in the progression of development and cataractogenesis. We found very interesting molecules, Tm1α, which is a target of miR29c (Figs 6), as demonstrated by protein expression analysis. These molecules have been reported to have impact in maintaining cell biology including cells of the eye lens [37, 53, 95]. This was the first demonstration that Tm1α is a target gene for miR29c, at least in lens. Lens cells express tropomyosin similar to that of erythrocytes [96]. However, little is known about how the expression levels of Tms are regulated during development and cataract formation, and how they are connected to cellular integrity. Tropomyosins are known to be associated with the regulation of actin cytoskeleton, and to play a role in LEC differentiation [37, 53]. Overstimulation of Tm isoforms and stress fibre, and thereby their involvement in pathogenic processes, has been suggested [97]. Recently, using SCR as a model system, we showed that the physiological level of Tms expression is vitally important for cellular homeostasis [37]. SCRs develop cataract spontaneously in the perinuclear and nuclear portions at 11–12 weeks of age [42]. Lens opacity in the SCR appears in 70% of animals, whereas the remainder have normal clear lenses (non-cataractous) [42]. This indicates that cataract formation in the SCR is an ‘all or none’ phenomenon. In our current study, we showed a pattern of expression of miRNAs in cataractous and non-cataractous lenses of SCR. We believe that outcome of this study will be helpful in dissecting out the roles of miRNAs in cataractogenesis.

Up-regulation of Tm1α and Tm2β proteins was observed in SCR with cataractous LECs. The increased expression of these molecules was inversely related to miR29 in LECs of SCRs with cataract. These novel findings were further verified by expression inhibition and overexpression experiments using miR29c inhibitor and precursor respectively. These experiments revealed that, indeed, Tm1α and Tm2β are targets of miR29c in lenses or LECs of SCRs without cataract and/or SCRs with cataract. These data imply that miR29c may directly or indirectly regulate the translation of Tm1α and Tm2β. In a previous study, we found that Prdx6-deficient (*Prdx6*^*−/−*^) LECs showed phenotypic changes and formed lentoid bodies, a characteristic of terminal cell differentiation and the epithelial-to-mesenchymal transition (EMT) [1]. This EMT occurred as a result of oxidative stress-induced activation and expression of TGFβ1 in *Prdx6*^*−/−*^ cells [1, 31, 34]. Prdx6 provides cytoprotection against internal and external environmental stresses and plays a role in cellular signalling by optimizing ROS and thereby controlling gene regulation [1, 33, 98, 99]. Furthermore, proteomic analysis of *Prdx6*^*−/−*^ LECs showed elevated expression of cytoskeleton proteins Tm1α and Tm2β [34]. Using *in vivo* rodent posterior capsule opacity and human cataractous LECs as model systems, we demonstrated overstimulation of Tm1α/2β. This result suggests that Tm1α and Tm2β are involved in remodelling the actin cytoskeleton during EMT of LECs [37]. However, aberrant expressions of Tms have been documented in initiation and progression of pathophysiology of tissues including cancer [45, 46]. We believe that optimizing the expression levels of Tms by manipulating the expression of miR29c may postpone or delay posterior cataract opacification (PCO), anterior subcapsular fibrosis (ASF) and severe nuclear cataracts resulting from overinduction of abnormal cellular changes such as fibrosis in LECs. This regulation of miR29c may have effects in other disorders associated with aberrant Tms expression [37]. The finding may open a path to establishing and developing miRNA-based therapeutics to ablate the development of anti-PCO or ASF therapies.

In summary, we have shown, for the first time, the temporal and spatial expression profiles of miRNAs in rat eye lens, and identified several miRNAs that are modulated with progression of development or during cataractogenesis. The loss of miRNA function during a disease state or abnormalities in the development process may be as a result of transcription repression, deletion, epigenetic silencing or aberrant expression of miRNA. Although further work is necessary for a more precise definition of the processes involved, this initial delineation of the level of miRNA expression in lens provides a beginning, along with our identification of the specific miR29c and its target gene, Tms.

Finally, a critical understanding of the role of miRNAs and their expression profiles should aid in identifying whether miRNAs. The specific gene targets of miRNAs may have application as diagnostic markers or clues to the prognosis for age-associated diseases such as cataractogenesis.
